# Hydrophobically-Modified PEG Hydrogels with Controllable Hydrophilic/Hydrophobic Balance

**DOI:** 10.3390/polym13091489

**Published:** 2021-05-06

**Authors:** Fabio Bignotti, Francesco Baldi, Mario Grassi, Michela Abrami, Gloria Spagnoli

**Affiliations:** 1 Department of Mechanical and Industrial Engineering, University of Brescia, via Branze 38, I-25123 Brescia, Italy; francesco.baldi@unibs.it (F.B.); gloria.spagnoli@unibs.it (G.S.); 2 Department of Engineering and Architecture, University of Trieste, Building B, via Valerio 6, I-34127 Trieste, Italy; mario.grassi@dia.units.it (M.G.); michela.abrami@dia.units.it (M.A.)

**Keywords:** hydrogels, epoxy, hydrophobic association, thermosensitive, pH-sensitive, amphiphilic, PEG, swelling behavior, mechanical properties, low field NMR

## Abstract

This work reports on a novel method to synthesize hydrophobically-modified hydrogels by curing epoxy monomers with amines. The resulting networks contain hydrophilic poly(ethylene glycol) (PEG) segments, poly(propylene glycol) (PPG) segments, and C_18_ alkyl segments. By varying the content of C_18_ segments, networks with different hydrophilic-lipophilic balance (HLB) are obtained. All networks show an amphiphilic behavior, swelling considerably both in organic solvents and in aqueous media. In the latter they display a thermosensitive behavior, which is highly affected by the network HLB and the pH of the solution. A decrease in HLB results in an increment of the polymer weight content (w_p_) due to hydrophobic association. Furthermore, a reduction in HLB induces a remarkable increase in initial modulus, elongation at break and tensile strength, especially when w_p_ becomes greater than about 10%. Low field nuclear magnetic resonance (LF-NMR) experiments evidence that, when HLB decreases, a sudden and considerable increase in hydrogel heterogeneity takes place due to occurrence of extensive physical crosslinking. Available data suggest that in systems with w_p_ ≳ 10% a continuous physical network superimposes to the pre-existing chemical network and leads to a sort of double network capable of considerably improving hydrogel toughness.

## 1. Introduction

Polymeric hydrogels are soft materials with a network structure that contains considerable amounts of water. They have found use mainly in biomedical applications, including contact lenses, hygiene products, wound dressings, drug delivery, or regenerative medicine [1]. Non-medical applications include sensors, agriculture, water treatment, and the food industry [2,3,4,5]. Chemically cross-linked hydrogels present a network of covalent bonds. Physically cross-linked networks are stabilized by reversible junctions such as hydrophobic association, hydrogen bonds, or crystalline domains. Hybrid systems contain in their structure both chemical and physical crosslinks [6].

Amphiphilic polymers contain both hydrophilic (HI) and hydrophobic (HO) moieties along their chain. Their ability to form micelles, rods, or vesicular aggregates makes them attractive candidates in drug delivery due to possibility of encapsulating drugs in these nano assemblies. The HLB of amphiphilic polymers greatly affects the nanoaggregate morphology and their performance at the biological interface [7,8].

Amphiphilic hydrogels, often referred to as hydrophobically modified (HM) hydrogels, have been widely investigated [9,10,11,12]. In addition to the physical crosslinks resulting from self-assembly, in some HM hydrogels chemical crosslinks can be present. The presence of HO and HI segments allows these systems to swell appreciably both in aqueous media and in organic solvents. The nanoaggregates deriving from the self-assembly of HO segments can absorb hydrophobic substances such as drugs. The extent of hydrophobic association changes with temperature, therefore, HM hydrogels have found application in the controlled release of pharmacological substances [13]. Furthermore, the presence of hydrophobic segments significantly improves their toughness in comparison with traditional hydrogels, which often feature poor mechanical strength. The reversible dissociation of hydrophobic associations allows to relieve mechanical stresses without loss of integrity, thus increasing hydrogel toughness [14,15].

HM hydrogels have been mainly synthesized using the free radical micellar copolymerization of hydrophobic and hydrophilic monomers in the presence of surfactants [14,16,17], which largely affect the association behavior and final properties of the resulting hydrogels [18,19]. Surfactants containing reactive groups have been employed to avoid surfactant removal [15]. Other methods employed in the preparation of HM hydrogels include grafting of HO segments on hydrophilic polymers [20,21] or the chemical combination of hydrophilic and hydrophobic prepolymers [22].

The aim of the present work is to report on a novel method to synthesize HM hydrogels. The method relies on the reaction of amine and epoxy monomers and allows to modify independently the chemical and physical crosslink density of the network. To the best of the authors’ knowledge, no previous report has been published on the use of epoxy monomers to prepare HM hydrogels. In this specific case the HI residues were poly(ethylene glycol) (PEG) and poly(propylene glycol) (PPG) segments while the HO blocks were C_18_ alkyl segments. PEG is an amphiphilic polymer used in many hydrogel preparations because of its nontoxicity and biocompatibility [23]. PPG is another amphiphilic polymer, though less hydrophilic than PEG, which is typically employed as food additive, in cosmetics or in the synthesis of biomedical polyurethanes [24]. The C_18_ alkyl segments were incorporated in the network using octadecylamine (ODA), a lipophilic amine recently used in liposomes to improve the bioavailability of poorly water-soluble drugs [25] and in gene delivery systems to enhance their transfection ability [26].

The networks here investigated exhibited an amphiphilic nature. Their room temperature swelling behavior was tested in six different organic solvents. In aqueous media the networks displayed a thermosensitive behavior, which was investigated between 8 °C and 70 °C at three different pHs. Low field nuclear magnetic resonance (LF-NMR) experiments were carried out to gather information on the extent of hydrophobic association in NaCl 0.1 M. Finally, the room temperature mechanical properties of the hydrogels were measured by uniaxial tensile tests, which were run on specimens equilibrated in 0.1 M NaCl.

## 2. Materials and Methods

### 2.1. Materials

Poly(ethylene glycol)diglycidyl ether (PEGDE), Jeffamine M600 (JM600), Jeffamine D400 (JD400), and ODA (97% purity) were purchased from Sigma-Aldrich Italy (Milan, Italy) and employed as received. The molar mass of PEGDE, determined according to ASTM D1652 [27] was 524 ± 1 g/mol. The molar mass of JM600 and JD400, determined by potentiometric titration with 0.1 M HCl, was 629 ± 4 g/mol and 469 ± 2 g/mol, respectively. All the above values of equivalent mass were obtained from an average of three measurements.

### 2.2. Synthesis of HM Hydrogels

PEGDE, JM600, and ODA were mixed in the desired ratio and heated at 80 °C under a nitrogen atmosphere. After one hour JD400 was added, then the reaction mixture was poured into silicone molds and oven cured for 45 h at 80 °C under nitrogen atmosphere. Rectangular specimens (90 × 7 × 2 mm^3^) were obtained which were soaked into isopropanol to remove the un-crosslinked fraction. Purification was carried out by changing the solvent at intervals and checking the pH till neutrality was reached (about four days). Then some specimens (dry specimens) were desiccated at 50 °C under vacuum up to constant weight. The other specimens (wet specimens) were soaked first into an isopropanol/0.1 M NaCl mixture (50/50 *v*/*v*) for 24 h and then in 0.1 M NaCl. The solvent was changed regularly till hydrogels reached the equilibrium volume (about six days).

### 2.3. FTIR Analysis

Fourier transform infrared (FT-IR) spectra were recorded on specimens obtained from evaporation of isopropanol (dry specimens) using a Thermo Fisher (Waltham, MA, USA) Nicolet iS50 spectrometer equipped with a PIKE MIRacle attenuated total reflectance attachment (diamond crystal). On each sample 64 scans were recorded from 600 to 4000 cm^−1^ at a resolution of 4 cm^−1^.

### 2.4. TGA Analysis

High-resolution thermogravimetric (TGA) analyses were performed using a TA Instruments (New Castle, DE, USA) Q500 thermogravimetric analyzer and an initial heating rate of 50 °C/min. A sample (ca. 15 mg) of dry specimen was heated in a 100 microliter open platinum pan from room temperature to 700 °C under a nitrogen atmosphere at a purge rate of 60 mL/min.

### 2.5. Swelling Tests

A strip (typical size: 10 × 7 × 2 mm^3^) of mass m_d_ was cut from dry specimens, allowed to equilibrate at 25 °C in the desired solvent and the mass of the swollen strip (m_s_) was measured. The swelling ratio (SR) and the polymer weight fraction (w_p_) were evaluated as:

(1) $$\mathrm{SR}=\frac{m_{s}}{m_{d}}$$

(2) $$w_{p}=\frac{1}{\mathrm{SR}}$$

The swelling tests in NaCl 0.1 M were carried out at different temperatures as follows. A strip was cut from a wet specimen and put at 5 °C. After 24 h its mass m_s_ was measured, then the temperature was increased step by step up to 70 °C weighing the strip after 24 h of equilibration at each temperature. Finally, the strip was soaked in distilled water to remove the salt, dried and weighed to determine its dry mass m_d_. The values of SR and w_p_ were evaluated at every temperature using Equations (1) and (2), respectively. All swelling measurements were repeated on four different strips.

### 2.6. LF-NMR Experiments

LF-NMR measurements were performed at 25 °C by a Bruker Minispec mq20 (0.47 T, Karlsruhe, Germany). according to the CPMG sequence (Carr–Purcell–Meiboom–Gill) [28] {90°[−τ − 180° − τ(echo)]n − TR} with a 8.36 μs wide 90° pulse, τ = 250 μs and a T_R_ (sequence repetition rate) equal to 5 s. Each spin-echo decay had n points and was repeated 36 times (number of scans).

The relaxation process was described by the sum of exponential terms each one characterized by a different time decay constant (T_2i_) and weight (A_i_) [29]:

(3) $$I(t)=\overset{m}{\underset{i=1}{\sum }}A_{i}e^{(-t/T_{2i})}$$

where I(t) is the dimensionless signal amplitude that becomes negligible at the end of the relaxation process. The average relaxation time (T_2m_) can be defined by:

(4) $$T_{2m}=\sum_{i=1}^{m}A_{i}T_{2i}/\sum_{i=1}^{m}A_{i}\;A_{i\%}{=100A}_{i}/\sum_{i=1}^{m}A_{i}$$

The number, m, of exponential decays appearing in Equations (3) and (4) was determined by a statistical procedure based on the minimization of the product (2 × N × χ) [30] where χ is sum of the squared errors and 2N is the number of model fitting parameters. The m couples (T_2i_, A_i%_) represent the relaxation time distribution.

### 2.7. Uniaxial Tensile Tests

Uniaxial tensile tests were performed at room temperature with an Instron (model 3366, Norwood, MA, USA) test system on hydrogels previously equilibrated in NaCl 0.1 M at 23 °C. The tests were carried out until fracture on bar specimens (gauge length: 40 mm; width ≈ 10 mm; thickness ≈ 3 mm) using a crosshead speed of 40 mm/min. At least four repetitions were carried out for each test. The mechanical behavior was evaluated in terms of nominal stress σ = F/A_0_ vs. percent elongation ε% = 100 × (Δl/l_0_) curves, where F is the recorded load, A_0_ the initial specimen cross-section, and l_0_ and Δl the initial gauge length and the gauge length extension, respectively. The initial modulus, E_in_, was determined as the slope of the straight-line tangent to the stress-strain curve at very small strains.

## 3. Results and Discussion

### 3.1. Hydrogel Preparation

**Figure 1:**
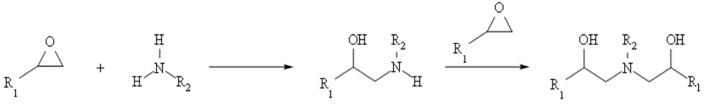
Reaction of a primary amine with an epoxy compound.

The procedure followed to prepare the HM hydrogels is based on the reaction of epoxy groups with amine hydrogens (see Figure 1), which is widely employed to cure epoxy resins [31]:

The functionality of monomers, that is the number of reactive groups present in their structure, plays a key role on the structure of the final product. If a diepoxide is reacted with a primary monoamine, an uncrosslinked comb-like polymer is obtained. Chemically-crosslinked networks are obtained when at least one of the reactants has a functionality higher than two. In that case, the theory on stepwise polymerizations [32] predicts that, in the absence of side reactions, the chemical crosslink density is maximum when the stoichiometric ratio (q):

(5) $${q=\mathrm{eq}}_{\mathrm{ep}}/{\mathrm{eq}}_{\mathrm{am}}$$

is unity and decreases as q deviates from unity. In the above equation “eq” represents the number of equivalents, namely the moles of epoxy groups (eq_ep_) or amine hydrogens (eq_am_) incorporated in the reaction mixture. Crosslinked systems can be obtained also when a diepoxide is reacted with a mixture of a monoamine and a diamine. In that case, the diamine is used as crosslinking agent while the monoamine allows to introduce dangling segments along the network strands.

**Figure 2:**
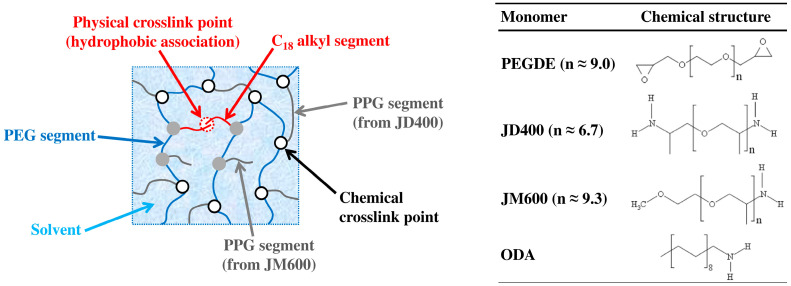
Schematic view of the structure of a HM hydrogel and chemical structure of the monomers.

In the present work, different networks were prepared by chemically crosslinking PEGDE, a diepoxide, with JD400, a bis-primary amine. Pendant segments were introduced in the network by using a mixture of JM600 and ODA, two primary amines. The structure of the monomers is reported in Figure 2.

Three parameters are needed to express the composition of the reaction mixture. In addition to the stoichiometric ratio q, the following parameters were employed:

(6) $$f_{\mathrm{JD}400}{=\mathrm{eq}}_{\mathrm{JD}400}/{\mathrm{eq}}_{\mathrm{am}}$$

(7) $$f_{\mathrm{ODA}}{=\mathrm{eq}}_{\mathrm{ODA}}/{(\mathrm{eq}}_{\mathrm{ODA}}{+\mathrm{eq}}_{\mathrm{JM}600})$$

where eq_JD400_, eq_ODA_, and eq_JM600_ are the number of aminic hydrogens deriving from JD400, ODA and JM600, respectively, while eq_am_ represents the total number of amine hydrogens. From the above equations it follows that the higher the value of f_JD400_ (for a given stoichiometric ratio) the higher the concentration of crosslinking agent (JD400) and the expected chemical crosslink density. The higher f_ODA_ the higher the concentration of C_18_ alkyl segments undergoing hydrophobic association in aqueous media and consequently the expected degree of physical crosslinking (Figure 2).

Table 1 summarizes the compositions investigated in this work. In a first set of compositions only f_ODA_ was changed to investigate the effect of network HLB and hydrophobic association while in the second set only f_JD400_ was modified to highlight the influence of the chemical cross-link density.

**Figure 3:**
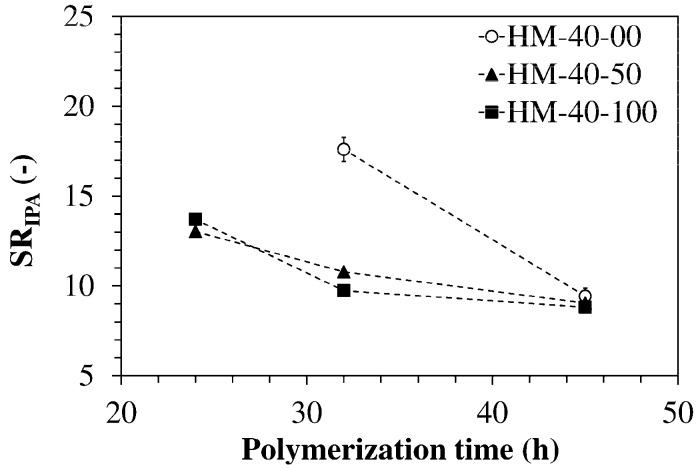
Variation of the equilibrium swelling ratio in IPA with the polymerization time at 80 °C (no crosslinking observed for HM-40-00 in 24 h). Each datum represents the average of four replicates.

In all cases an excess of amine over epoxy was employed (q < 1) to avoid the presence of residual epoxy groups that could react with foreign compounds or other reactive groups (hydroxy or amine groups) present in the network and lead to a progressive change in hydrogel structure over time. Neither solvents nor surfactants were used. The temperature of 80 °C was selected because preliminary experiments carried out at higher temperatures (100 and 120 °C) showed that the resulting hydrogels were very brittle and could even crumble during the purification step. Three compositions (HM-40-00, HM-40-50, and HM-40-100) were polymerized at 80 °C for 24 h, 32 h, or 45h to select a suitable polymerization time. Figure 3 shows that the SR of the networks containing ODA decreases with the polymerization time.

According to the Flory–Rehner theory [33], this indicates an increment of their crosslink density. The ODA-free system does not crosslink in 24 h and after 32 h its SR is much larger than that of the other two networks, suggesting that JM600 has a lower reactivity than ODA. This could depend in part on its greater bulkiness and in part on the presence of ether groups in its structure. In fact, epoxide-amine reactions are accelerated in the presence of hydrogen donors like hydroxy groups while they are decelerated by hydrogen bond acceptors like ether groups [31]. Despite the difference in monomer reactivity, the three networks reached similar values of swelling ratio after 45 h at 80 °C, thus, these were the conditions adopted for polymerizing all reaction mixtures.

**Figure 4:**
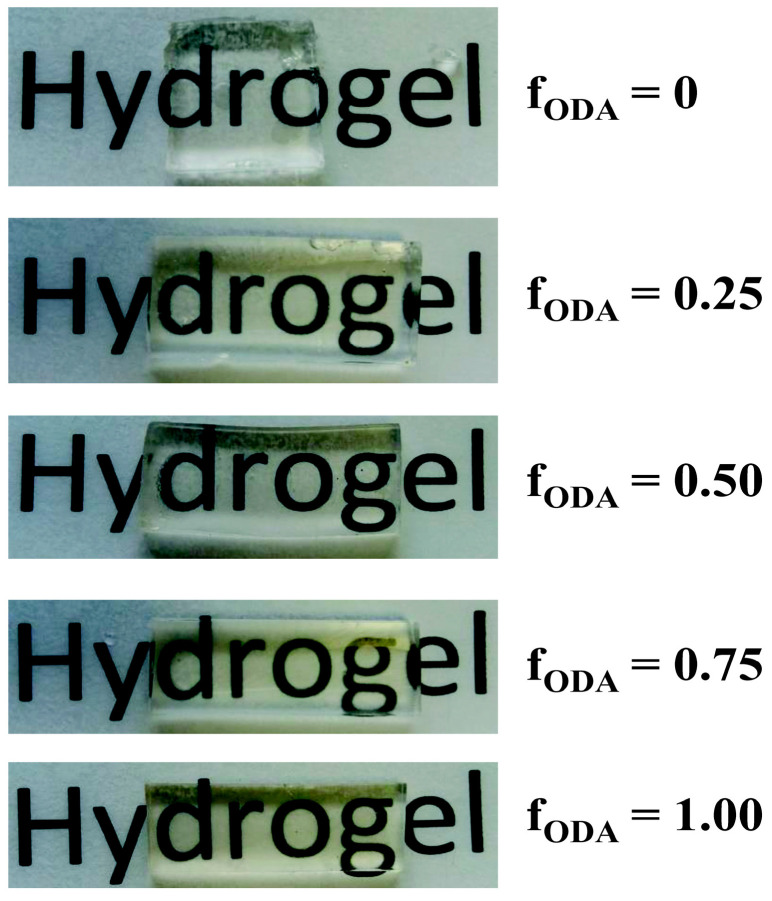
Images of hydrogel samples placed over the label “hydrogel”. Samples equilibrated in 0.1 M NaCl (thickness ca. 3 mm).

The reaction mixtures containing ODA were initially opaque due to its insolubility in the other monomers, but they became gradually transparent as the reaction proceeded. The resulting networks displayed good clarity, which further increased after equilibration in NaCl 0.1 M (Figure 4).

Their transparency suggests that even in the hydrogels containing the highest amount of ODA the in-homogeneities are smaller than the wavelength of light in the visible region (400–700 nm).

**Figure 5:**
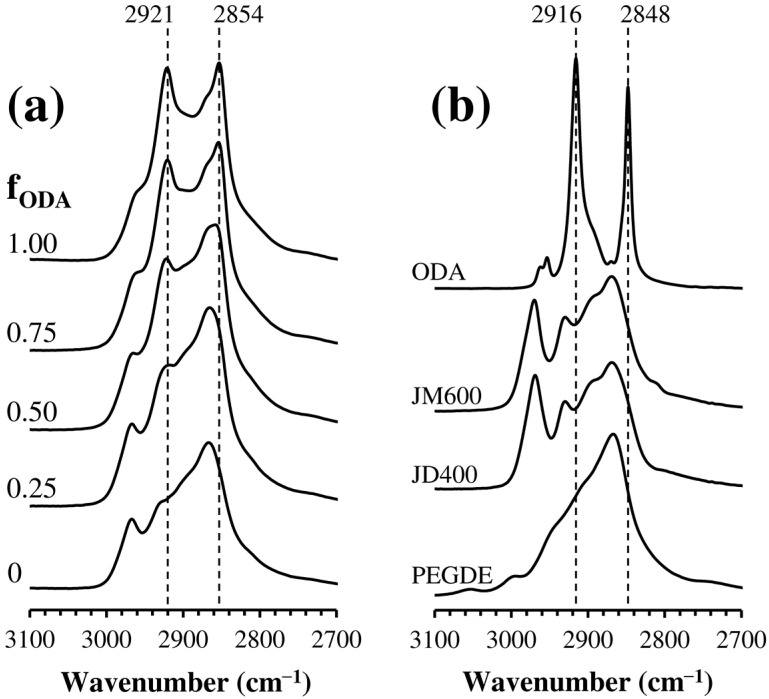
FTIR spectra in the region near 3000 cm^−1^ of (**a**) dry gels (f_JD400_ = 0.40) and (**b**) starting monomers.

Figure 5a shows the FTIR spectra of dry gels. The intensity of the peaks near 2920 cm^−1^ and 2850 cm^−1^, which are ascribed to the stretching of C-H bonds in methylene ODA groups (see Figure 5b), increases with f_ODA_. Therefore, the network HLB decreases as the ODA content in the reaction mixture is increased.

**Figure 6:**
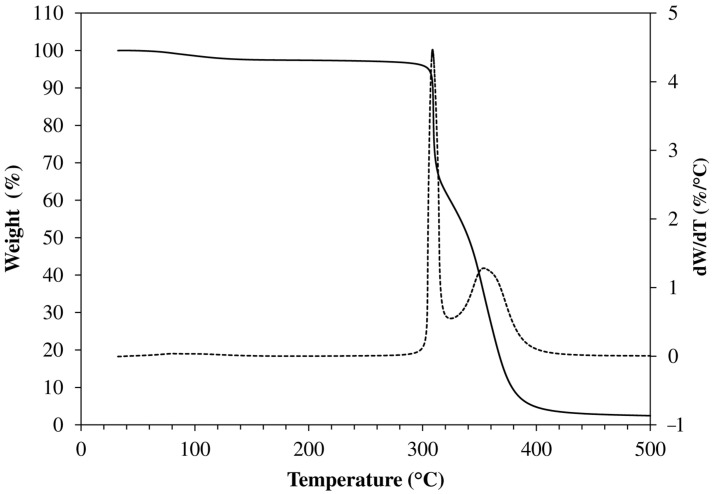
Typical traces obtained in high resolution TGA experiments carried out under nitrogen (sample HM-40-50).

Samples of dry gels were analyzed by TGA to gather more quantitative information about their composition. As shown in Figure 6, the TGA traces exhibited a small initial weight loss (3–4%), probably due to the evaporation of residual solvent and/or moisture, and two main weight loss steps, the former at 300–320 °C and the latter at 350–360 °C. The occurrence of a weight loss near 315 °C and 345 °C has been reported for poly(propylene oxide) and poly(ethylene oxide), respectively [34].

A summary of TGA data is reported in Table 2. Almost in all cases, the weight loss of the second step (WL_2_) is in excellent agreement with the PEGDE content in the corresponding feed mixture (Table 1). Only for f_ODA_ = 1 the PEGDE content in the reaction mixture is remarkably higher (5.2%) than WL_2_. 

The first weight loss can be ascribed to the volatilization of JD400, JM600 and ODA residues. The difference between WL_1_ and their content in the feed mixture ranges between 0% (HM-40-100) and 6.2% (HM-40-00). Therefore, it can be reasonably concluded that the composition of the networks is not dissimilar from that of the reaction mixture.

### 3.2. LF-NMR Analysis

Table 3 reveals that when ODA is absent (f_ODA_ = 0; HM-40-00) only one relaxation time characterizes the polymeric network, this indicating the existence of a homogeneous structure where all the hydrogen atoms experience the same magnetic relaxation conditions. This translates into a polymeric network showing a homogeneous distribution of the crosslink density throughout the whole hydrogel volume.

On the contrary, when ODA is present (f_ODA_ = 0.25 − 1; HM-40-25 to HM-40-100), the relaxation spectrum is characterized by two components, the first of which (A_1_(%)—T_21_) associated to a higher relaxation time and the second one associated to a smaller relaxation time (A_2_(%)—T_22_). This clearly indicates that the polymeric network is heterogeneous as two different relaxation modes exist, and this reflects in a not homogeneous distribution of the crosslink density inside the network. As the smaller the relaxation time, the higher the crosslink density [35,36], we can conclude that zones characterized by higher (A_2_(%)—T_22_) and smaller (A_1_(%)—T_21_) crosslink density coexist inside the polymeric network. Interestingly, ODA increase implies the reduction of the average relaxation time T_2m_ as the fraction of higher crosslink density zones increases from 4% up to 96% (see Table 3).

**Figure 7:**
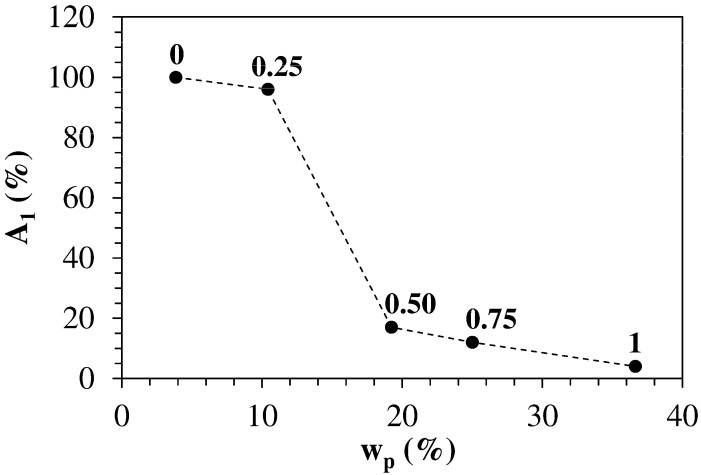
Variation of the fraction of the smaller crosslink density zones (A_1_(%)) with the polymer mass fraction w_p_, which is proportional to the reported values of f_ODA_. Each datum represents the average of 36 replicates.

In addition, the variation of the fraction of the smaller crosslink density zones, indicated by A_1_(%) (see Figure 7), is peculiar as it undergoes an abrupt reduction in the range 10% ≲ w_p_ ≲ 15%, corresponding to 0.25 < f_ODA_ < 0.50. This witnesses a sudden and important increase of the hydrogel heterogeneity imputable to the formation of physical crosslinks, induced by hydrophobic interactions, that superimpose to the pre-existing chemical crosslinks. In other words, up to w_p_ = 10% (f_ODA_ = 0.25), the crosslink density is essentially of chemical origin while the physical crosslink density becomes more and more important as the network HLB decreases.

### 3.3. Room Temperature Swelling Behavior

**Figure 8:**
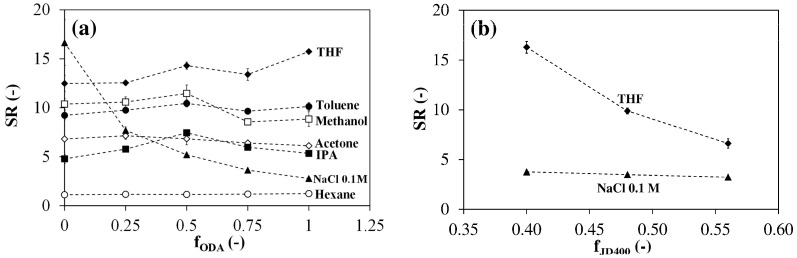
(**a**) Room temperature swelling ratio in various solvents as a function of f_ODA_ (q = 0.80; f_JD400_ = 0.40). (**b**) Effect of f_JD400_ on the room temperature swelling ratio in THF and in NaCl 0.1 M (q = 0.80; f_ODA_ = 0.75). Each datum represents the average of four replicates.

Dry specimens were immersed in different solvents and allowed to absorb the solvent until equilibrium was reached (about 96 h). Three sets of organic solvents were selected. Methanol and isopropanol are protic polar solvents able to develop strong hydrogen bonds and can act as hydrogen-bond donors. Acetone and THF are polar solvents with a moderate hydrogen-bonding tendency but are not hydrogen-bond donors. Hexane and toluene are low polarity aprotic solvents with no or low tendency to form hydrogen bonds. Figure 8a shows that all networks swell considerably in these solvents except in hexane, which possesses the lowest polarity and hydrogen bond ability.

Solubility tests performed at room temperature on starting monomers (c = 2 wt%) showed that PEGDE is insoluble only in hexane, JM600 and JD400 are soluble in all solvents while ODA is soluble only in THF. This suggests that the swelling behavior in organic solvents is mainly influenced by the PEG segments, which in all systems account for about 50–60 wt% of the network. While in organic media the swelling ratio is not much affected by the network HLB, in NaCl solution it decreases very quickly as f_ODA_ increases due to the association of an increasing number of C_18_ alkyl segments. Therefore, the swelling ratio in NaCl 0.1 M can be considered as a qualitative measure of hydrophobic association (physical crosslinking). Only in hexane all networks swell less than in NaCl 0.1 M. In the other cases, when f_ODA_ is sufficiently high the network swells more in organic solvents than in saline solution while the opposite takes place at low f_ODA_ values. Therefore, by adjusting the network HLB it is possible to minimize volume changes when passing from an organic solvent to aqueous media or vice versa.

Swelling data in organic solvents can provide qualitative information about the chemical cross-link density. Figure 8b shows that, when the concentration of the crosslinking agent in the feed mixture is increased, the swelling ratio remarkably decreases in THF whereas it remains almost constant in NaCl 0.1 M. THF is a good solvent for all the networks considered, as it can be inferred from the corresponding high SR values. In the light of the Flory-Rehner theory it is thus reasonable to conclude that the reduction observed in THF derives from an increase in the chemical crosslink density. In NaCl 0.1 M the swelling ratio is only slightly affected by f_JD400_ because physical crosslinking compensates for the effect of chemical crosslinking.

### 3.4. Thermo- and pH-Sensitivity in Aqueous Media

PEG is highly soluble in water below 80–90 °C. Low molecular weight PPG exhibits a lower critical solution temperature between 15 °C and 42 °C depending on its molecular weight [37]. PEG-PPG-PEG triblock copolymers (poloxamers), commercially known as Pluronics^®^, Synperonics^®^ or Lutrol^®^, are amphiphilic polymers employed in drug delivery, gene delivery or tissue engineering [38,39,40]. Poloxamers can form micelles above a critical concentration, which is temperature dependent [41]. When heated, the micelles form a 3D network and a sol-gel transition (thermogelling) is eventually observed provided their concentration is sufficiently high. Since the HM hydrogels investigated are cross-linked copolymers containing PEG and PPG segments, their swelling behavior in aqueous media was studied as a function of temperature.

**Figure 9:**
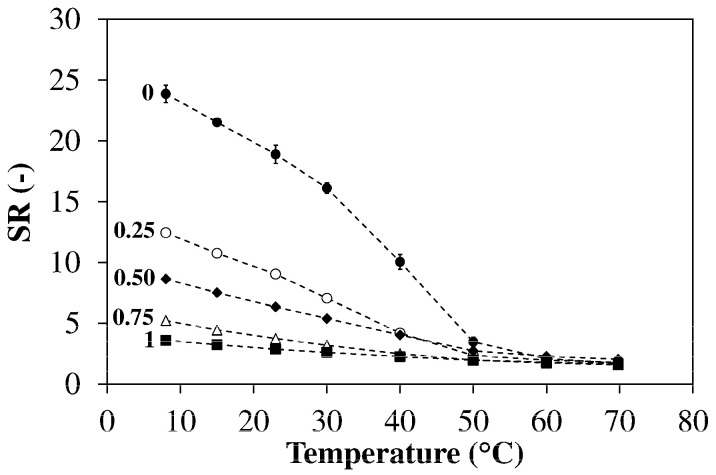
Temperature dependence of the swelling ratio in NaCl 0.1 M. Hydrogels prepared using q = 0.80, f_JD400_ = 0.40 and the reported values of f_ODA_. Each datum represents the average of four replicates.

Figure 9 shows the dependence of SR on temperature for hydrogels with different ODA contents. At low temperature (8 °C) the SR decreases with f_ODA_ from 24 (f_ODA_ = 0) to 3.6 (f_ODA_ = 1). When the temperature increases, all hydrogels shrink appreciably. In the absence of ODA, a volume transition is clearly visible above 40 °C and SR reaches a value of 1.7 at 70 °C. As the network HLB decreases the transition tends to shift to lower temperatures and become less evident. This trend could be explained using the mechanism proposed to interpret thermosensitivity in poloxamers [42].

At low temperature both PEG and PPG segments are hydrated but, when the temperature is increased, the PPG segments become dehydrated because of their higher hydrophobicity. We suggest that the dehydration of PPG segments and expulsion of water determine the considerable shrinking observed in HM-75-00 (f_ODA_ = 0). As f_ODA_ increases, the PPG content within the polymer network is progressively reduced from 55 wt% (HM-75-00) to 14 wt% (HM-75-100) causing a reduction in hydrogel thermosensitivity. Moreover, the hydrophobic association of C_18_ alkyl segments reduces the SR in the low temperature range, thus further decreasing the thermosensitivity.

**Figure 10:**
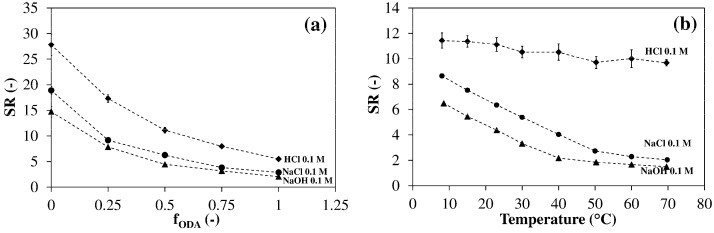
Effect of the pH on the swelling behavior. (**a**) Dependence of the room temperature swelling ratio from f_ODA_. (**b**) Swelling ratio vs. temperature for the hydrogel with f_ODA_ = 0.50. Each datum represents the average of four replicates.

From Figure 1 it is evident that the networks investigated in the present work contain amino groups, which are weak bases and, therefore, possess a degree of protonation dependent from the pH of the solution. It is, thus, expected that HM hydrogels display pH-sensitivity [43]. Figure 10a shows that in 0.1 M NaOH (pH ≈ 13), where all amino groups are deprotonated, all hydrogels possess the lowest degree of swelling. In 0.1 M HCl (pH ≈ 1) all nitrogens are protonated. The electrostatic repulsion of charged groups leads to an expansion of the network, therefore, the highest values of SR are observed. An intermediate behavior is noticed at pH ≈ 7 (0.1 M NaCl), where the amino groups are only partly protonated. In Figure 10b the effect of temperature at the same pHs is compared.

In 0.1 M HCl the repulsion between the charged groups is so high that it almost completely balances the effects of the hydrophobic association. Only a small (15%) linear decrement in SR is observed passing from 8 °C to 50 °C. In the same range of temperature, a 70% decrease in the SR ratio occurs in the other media, while the shrinking rate above 50 °C is similar to that observed in HCl. The volume transition is shifted to lower temperatures in NaOH 0.1 M compared to NaCl 0.1 M because of the lower degree of protonation of amino nitrogens at pH 13.

### 3.5. Tensile Behavior

The mechanical behavior of hydrogels was investigated using uniaxial tensile tests, which were carried out in air on hydrogels previously equilibrated at 23 °C in 0.1 M NaCl. A summary of tensile properties is reported in Table 4.

All materials fractured at large strains and recovered the whole deformation, thus featuring a rubber-like behavior, but different trends were observed when the chemical or physical cross-link density were changed.

**Figure 11:**
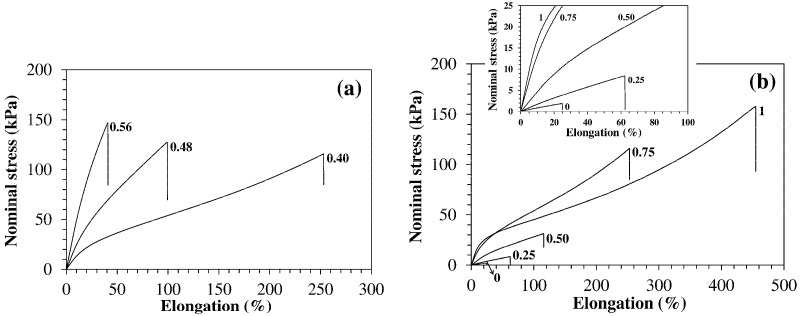
Stress vs. strain behavior of HM hydrogels (tests run in air on specimens equilibrated in NaCl 0.1 M, T = 23 °C). (**a**) Hydrogels synthesized using f_ODA_ = 0.75 and the indicated values of f_JD400_. (**b**) Hydrogels synthesized using f_JD400_ = 0.40 and the indicated values of f_ODA_. Inset: Initial portion of the stress-strain curves.

Typical stress versus elongation curves of hydrogels prepared using the same f_ODA_ value (0.75) but different values of f_JD400_ are shown in Figure 11a. These systems have similar HLB but their chemical cross-link density (ν_chem_) increases with f_JD400_. When f_JD400_ increases from 0.40 to 0.56 the initial modulus changes from 140 kPa to 540 kPa, whereas the elongation at break drops from 260% to 35%.

According to the rubber elasticity statistical theory, the initial modulus of unfilled elastomeric networks increases proportionally to ν_chem_ [33]. Conversely, the stretch ratio at break λ_b_ = l_b_/l_0_ (l_b_ and l_0_ are the length of the gauge at break and in the undeformed state) is expected to decrease proportionally to ν_chem_^−1/2^ [44]. Thus, this theory predicts that, if the only parameter changed is ν_chem_, an increment in mechanical stiffness can only occur at the expenses of a reduction in extensibility. The results of Figure 11a and Table 4 are in qualitative agreement with these predictions. A different trend is observed when the network HLB is changed while keeping constant f_JD400_ and, therefore, ν_chem_ (Figure 11b).

The ODA-free hydrogel is a very weak material with an initial modulus of 8 kPa, an elongation at break of 26% and a stress at break of 1.6 kPa. As the network hydrophobicity increases, all these properties show a considerable increment up to 180 kPa, 450%, and 160 kPa for f_ODA_ = 1, respectively. As a result, the modulus of toughness, i.e., the area under the stress-strain curve up to the break point, is dramatically increased. HM-40-00 contains only covalent cross-links. Hydrogels of this type usually show poor toughness. They relieve mechanical stresses mainly by chain rupture. The irreversible nature of this process, combined with cross-link density inhomogeneities, makes them highly susceptible to fracture [45]. If hydrogels contain physical crosslinks like polyion complexes [46], hydrogen bonds [47], or hydrophobic associations [14,15,17,48], such crosslinks can dissociate when a stress is applied and re-form when the stress has relaxed. This process provides an energy dissipation mechanism able to hinder the propagation of cracks and improve their toughness [16,49].

**Figure 12:**
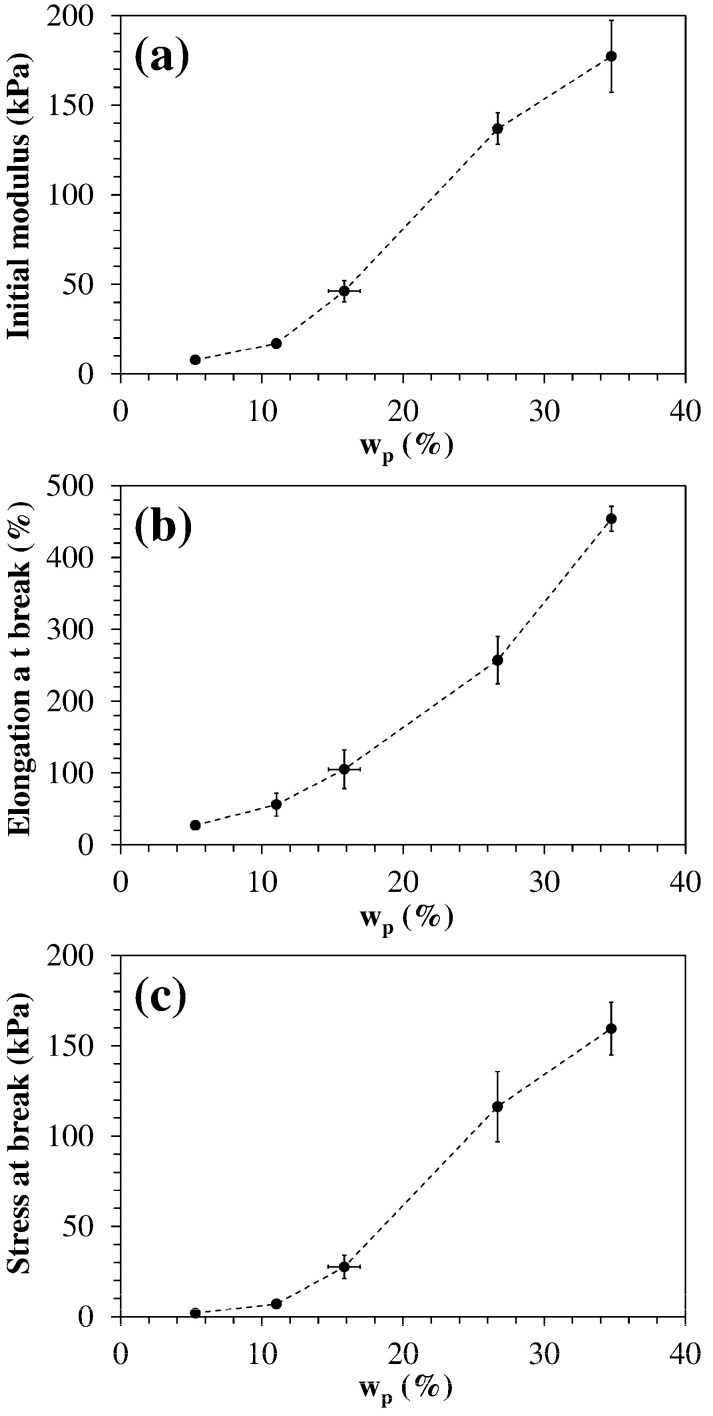
Variation of (**a**) initial modulus, (**b**) elongation at break, and (**c**) stress at break with the polymer weight content (f_JD400_ = 0.40, f_ODA_ from 0 to 1). Each datum represents the average of four replicates.

Figure 12 shows the dependence of tensile properties from the polymer weight fraction. At low values of w_p_ the properties increase rather slowly, but their increment becomes more pronounced when w_p_ ≳ 10%.

This trend could be explained by considering that higher HLB hydrogels have a low concentration of C_18_ alkyl segments able to provide mechanical reinforcement through physical crosslinking. When the HLB decreases and the concentration of C_18_ segments exceeds a critical value (C_crit_) they can form a continuous physical network in the same way as un-crosslinked amphiphilic polymers self-associate in water and form physical gels above a critical concentration [50]. This hypothesis is confirmed by the LF-NMR characterization that indicates how the fraction of lower crosslink density zones, proportional to A_1_ (%), decreases with w_p_, while higher crosslink density zones, proportional to A_2_ (%)—(see Table 3 and Figure 7), increase with w_p_.

Low HLB hydrogels somewhat resemble the so-called double-network hydrogels developed by Gong and co-workers [51], which consist of two chemically crosslinked networks, one tightly crosslinked and the other loosely crosslinked. According to a model proposed by Brown [49], double-network hydrogels show high toughness because, when cracks form in the tightly crosslinked network, they are held together by the second network, so extra energy is needed to propagate the cracks. Similarly, the reason for the high increment in toughness observed in the present case above C_crit_ could be the extra energy consumed to break the physical network in the regions near the crack tip.

## 4. Conclusions

This work shows that the chemistry of epoxy resins can be exploited to synthesize hydrogels with chemical and physical cross-link densities that can be independently varied. The former can be changed by modifying the monomer functionality while the latter can be modified by changing the network HLB and, therefore, the extent of hydrophobic association. The materials investigated swell considerably in both organic solvents and water, displaying an amphiphilic behavior. In aqueous media all hydrogels exhibit a thermo- and pH-sensitive behavior that becomes more evident as the network HLB increases. As the network HLB decreases due to an increasing content of C_18_ alkyl segments, the room temperature polymer content w_p_ increases because of the increased hydrophobic association. A considerable improvement in mechanical stiffness, strength, and toughness is observed in lower HLB hydrogels with w_p_ ≳ 10%. Low field NMR experiments suggest that in such systems zones with different crosslink densities co-exist and a drastic increment in physical crosslinking (hydrophobic association) occurs. It is, thus, suggested that the observed enhancement of mechanical properties derives from the presence of a double network consisting of a continuous physical network that superimposes to the network of covalent bonds.

## Figures and Tables

**Table 1:** Composition of the feed mixtures used to prepare HM hydrogels.

Code	q	f_JD400_	f_ODA_	ODA (wt%)	JM600 (wt%)	JD400 (wt%)	PEGDE (wt%)
HM-40-00	0.80	0.40	0.00	0	42.4	10.5	47.1
HM-40-25	0.80	0.40	0.25	4.8	33.8	11.2	50.2
HM-40-50	0.80	0.40	0.50	10.3	24.1	12.0	53.6
HM-40-75	0.80	0.40	0.75	16.5	13.0	12.9	57.6
HM-40-100	0.80	0.40	1.00	23.9	0	13.9	62.2
HM-48-75	0.80	0.48	0.75	14.6	11.3	15.7	58.4
HM-56-75	0.80	0.56	0.75	12.5	9.8	18.5	59.2

**Table 2:** Summary of high resolution TGA data.

Code	Initial Weight Loss (%)	T_peak1_^a^ (°C)	WL_1_^b^ (%)	T_peak2_^a^ (°C)	WL_2_^b^ (%)	Residue at 700 °C (%)
HM-40-00	3.1	301	46.7	355	48.0	1.8
HM-40-25	3.5	311	44.6	352	49.2	2.5
HM-40-50	2.9	307	40.7	353	53.6	3.5
HM-40-75	2.2	312	40.3	354	55.5	1.6
HM-40-100	2.4	318	37.8	353	57.0	2.0
HM-48-75	3.6	308	37.9	358	56.6	1.0
HM-56-75	2.8	309	36.6	358	58.4	1.7

^a^ Temperature corresponding to the first (T_peak1_) or second (T_peak2_) peak of dw/dT. ^b^ Weight loss of the first (WL_1_) or second (WL_2_) volatilization step.

**Table 3:** Summary of the relaxation spectra (A_i_(%)—T_2i_) characterizing the studied hydrogels. T_2m_ indicates the average relaxation time (water relaxation time at 25° is ≈ 3000 ms).

Code	f_ODA_	T_2m_ (ms)	T_21_ (ms)	A_1_ (%)	T_22_ (ms)	A_2_ (%)
HM-40-00	0.00	2332	2332	100	-	0
HM-40-25	0.25	1415	1460	96	191	4
HM-40-50	0.50	861	1607	17	709	83
HM-40-75	0.75	571	1931	12	384	88
HM-40-100	1.00	204	695	4	186	96

**Table 4:** Summary of the tensile properties of hydrogels equilibrated in 0.1 M NaCl (23 °C).

Code	q	f_JD400_	f_ODA_	w_p_ (%)	E_in_^a^ (kPa)	ε_b_^b^ (%)	σ_b_^c^ (kPa)
HM-40-00	0.80	0.40	0.00	5.3 ± 0.2	8 ± 1	26 ± 6	1.6 ± 0.5
HM-40-25	0.80	0.40	0.25	11.1 ± 0.2	17 ± 2	60 ± 15	8 ± 2
HM-40-50	0.80	0.40	0.50	16 ± 1	45 ± 5	100 ± 30	26 ± 6
HM-40-75	0.80	0.40	0.75	26.7 ± 0.1	140 ± 10	260 ± 30	120 ± 20
HM-40-100	0.80	0.40	1.00	34.8 ± 0.1	180 ± 20	450 ± 20	160 ± 15
HM-48-75	0.80	0.48	0.75	28.8 ± 0.2	260 ± 20	90 ± 10	123 ± 7
HM-56-75	0.80	0.56	0.75	31.1 ± 0.1	540 ± 30	35 ± 5	145 ± 15

^a^ Initial modulus. ^b^ Elongation at break. ^c^ Stress at break.

## Data Availability

Data is contained within the article.
